# Genetic basis of the early heading of high-latitude weedy rice

**DOI:** 10.3389/fpls.2022.1059197

**Published:** 2022-12-05

**Authors:** Zhuan Li, Rui Gui, Xiaoyu Yu, Chengwei Liang, Juan Cui, Xue Zhao, Xuemin Zhang, Pengcheng Yu, Wenfu Chen, Jian Sun

**Affiliations:** Rice Research Institute, Shenyang Agricultural University, Shenyang, China

**Keywords:** GWAS, genetic resources, heading date, weedy rice, QTL mapping

## Abstract

*Japonica* rice (*Oryza sativa* L.) is an important staple food in high-latitude regions and is widely distributed in northern China, Japan, Korea, and Europe. However, the genetic diversity of *japonica* rice is relatively narrow and poorly adapted. Weedy rice (*Oryza sativa f. spontanea*) is a semi-domesticated rice. Its headings are earlier than the accompanied *japonica* rice, making it a potential new genetic resource, which can make up for the defects of wild rice that are difficult to be directly applied to *japonica* rice improvement caused by reproductive isolation. In this study, we applied a natural population consisting of weedy rice, *japonica* landrace, and *japonica* cultivar to conduct a genome-wide association study (GWAS) of the heading date and found four loci that could explain the natural variation of the heading date in this population. At the same time, we developed recombinant inbred lines (RILs) crossed by the early-heading weedy rice WR04-6 and its accompanied *japonica* cultivar ShenNong 265 (SN265) to carry out a QTL mapping analysis of the heading date and mapped four quantitative trait locus (QTLs) and three epistatic effect gene pairs. The major locus on chromosome 6 overlapped with the GWAS result. Further analysis found that two genes, *Hd1* and *OsCCT22*, on chromosome 6 (Locus 2 and Locus 3) may be the key points of the early-heading character of weedy rice. As minor effect genes, *Dth7* and *Hd16* also have genetic contributions to the early heading of weedy rice. In the process of developing the RIL population, we introduced fragments of Locus 2 and Locus 3 from the weedy rice into super-high-yielding *japonica* rice, which successfully promoted its heading date by at least 10 days and expanded the rice suitable cultivation area northward by about 400 km. This study successfully revealed the genetic basis of the early heading of weedy rice and provided a new idea for the genetic improvement of cultivated rice by weedy rice.

## Introduction

*Japonica* rice (*Oryza sativa* L.) is a staple food for most people in China, particularly in the northeast. It is crucial for economic development and ensuring global and national food security (Mao et al., 2017). Superior *japonica* varieties have been applied over a wide range for a long time. However, their long-term application will reduce genetic diversity (Wang et al., 2014). Due to genetic bottlenecks, new improvement strategies have encountered challenges.

Although wild rice resources have been widely suggested for rice improvement, the unfavorable characteristics of the hybrid offerings of wild rice and *japonica* rice far exceed the favorable characteristics due to reproductive isolation and geographic and genetic distance. Thus, wild rice is difficult to apply in breeding programs (Brambilla and Fornara, 2013; Brar and Khush, 2018; Xie et al., 2019).

As a semi-domesticated genetic resource, weedy rice (*Oryza sativa f. spontanea*) has no reproductive isolation with accompanied cultivar and adapts to the current paddy field ecosystem. In addition, weedy rice also possesses some adaptive alleles that cultivars lack. Among these alleles, the early-heading genes have great application potential for improving *japonica* cultivars, while the underlying molecular genetic mechanisms of the early heading in weedy rice are still not revealed, which hinders its use in breeding applications (Zhao et al., 2018; Sun et al., 2019).

With the rapid development of next-generation high-throughput sequencing technology and multi-omics, many genes related to the rice heading have been identified. The identification of genes related to photoperiodic pathway and analysis of their molecular regulatory network mechanisms can not only reveal the genetic variation in the rice heading but also guide the genetic improvement of rice breeding, which is of great significance and necessary to the high-yield breeding of rice (Ebana et al., 2011; Brambilla and Fornara, 2013; Shrestha et al., 2014; Hori et al., 2015; Onogi et al., 2016). Rice has two known relatively conserved photoperiodic flowering pathways: the *Hd1*-centered gene and its related genes under short-day (SD) conditions and the *Ehd1*-centered gene and its regulatory genes under long-day (LD) conditions (Li et al., 2015; Chen et al., 2022).

So far, many heading genes have been identified; however, the reason for weedy rice heading earlier than their accompanied cultivars is still largely unknown. We performed a genome-wide associated study (GWAS) and QTL mapping to reveal this genetic basis in this study. Meanwhile, we provide new genetic materials and gene resources for breeding early-heading and widely adapted *japonica* cultivated rice.

## Materials and methods

### Plant materials and growth conditions

A total of 274 accessions, including 154 modern *japonica* cultivars, 87 *japonica* landrace, and 33 *japonica* weedy rice at Asian high latitudes (WRAH), were planted in the paddy field in three consecutive years from 2019 to 2021 for GWAS of the heading date. Another recombinant inbred line (RIL) population of 165 individuals derived from the weedy rice WR04-6 and *japonica* cultivar ShenNong 265 (SN265 called super rice) was used for QTL mapping of the heading date. All materials were planted at Shenyang Agricultural University (123°25′E, 41°48′N, Liaoning Province, China, temperate semi-humid continental climate, under natural long day-length conditions, average day length >14 h from May to September, average temperature of 23.5°C, fertilizer applied in the field with the following standards: urea 300 kg/hm^2^, diammonium 220 kg/hm^2^, potassium chloride 220 kg/hm^2^). Each variety was planted in three rows with 1.2-m row length, 30 cm apart between rows, and 12-cm space in rows, with one plant per hill. Field management followed normal agricultural practices. The list of accessions used in this study is displayed in **Supplementary Tables S1, S2**.

### Phenotyping and statistical analysis

The first panicle beyond flag leaf 1 cm was recorded as the heading. The heading date was calculated as the date from sowing to the heading of half of the accession. The phenotype data are displayed in **Supplementary Tables S1 and S2**. Microsoft Excel 2019 and GraphPad Prism 9.0 were used for statistical analysis. The heading date difference of three ecotypes was tested by one-way ANOVA multiple comparisons. The D’Agostino–Pearson test method was used for the normal distribution test of the RIL population.

### Genome-wide association study

The genotype of the GWAS panel for the heading date has been effectively used to study the genetic basis of agronomic traits in weedy rice (Sun et al., 2022). All raw reads were screened for high quality with Q20 quality scores >95% and Guanine and cytosine (GC) content <50%. The reads of each accession were aligned and then mapped to the reference genome (IRGSP1.0) using BWA software. We used samtools v0.1.19 and GATK v4.0 for population SNP variant data, and potential PCR duplicates were removed. It is also necessary to control the single nucleotide polymorphism (SNP) quality with a minor allele frequency >0.05 and no more than 50% missing data. Finally, we got a high-quality SNP haplotype map with an average coverage depth of 23.2 times for each sample to proceed with follow-up analysis **(** **Supplementary Figure S1**). We performed the GWAS based on 1,311,445 genetic markers and a heading date of 3 years by using EMMAX software to fit a linear mixed model. To control the population structure during the GWAS, the first two principal components of the principal component analysis (PCA) were used as covariates. The Manhattan plots of GWAS results were drawn by R package CMplot. The threshold for genome-wide significance was determined by 10^-5^ and 10^-6^.

### Linkage disequilibrium block analysis and candidate gene association analysis

Based on the result of the GWAS, we performed linkage disequilibrium (LD) block analysis for the 2MB region surrounding the leading SNP. The LD block was defined by LDblockShow software. The genes located within the high LD blocks were selected for checking the gene annotation (https://rapdb.dna.affrc.go.jp/). Then, a haplotype-based association analysis for the genes within the LD block was conducted by candihap software (Li et al., 2020).

### Genotyping for genetic map construction and quantitative trait locus (QTL) mapping

DNA was extracted from fresh leaves of RILs by the Cetyltrimethylammonium Bromide (CTAB) method. The genotypes were obtained using a 50K liquid-phase sequence capture chip (Guo et al., 2021). According to the genotyping results, 13,097 polymorphic sites were used for genetic map construction. Finally, a linkage map containing 2,275 bins was constructed based on the 13,097 polymorphic sites, spanning a total genetic distance of 2,068.6 cM, and the average genetic distance between adjacent markers was 0.91 cM. Marker information for the genetic map constructed is listed in **Supplementary Table S3**. This linkage map was used for QTL mapping of the heading date (**Supplementary Figure S2**).

### QTL mapping for RILs

The heading date of the RIL population was used as phenotype data combined with a high-quality bin marker map to proceed with QTL mapping by IciMapping software with a threshold Likelihood of Odd (LOD) value of 2.5. Two models were applied in this study: inclusive composite interval mapping with an additive effect (ICIM-ADD) and inclusive composite interval mapping with an epistasis effect (ICIM-EPI).

### Molecular marker-assisted selection for breeding improvement

In developing RILs derived from WR04-6 and SN265, individuals with the early heading and other elite characteristics were selected. We extracted these individuals’ DNA by the CTAB method and designed primers by https://primer3.ut.ee/to detect the locus responsible for the early heading. Primers used in this study are displayed in **Supplementary Table S6**.

### Haplotype network analysis

In order to further reveal the domestication and evolution relationship of *Hd1* in different rice ecotypes, we used the sequencing data of 1,293 accessions to perform haplotype network analysis including five ecotypes, temperate *japonica*, tropical *japonica*, *japonica* intermediate, weedy rice, and wild rice [*Oryza rufipogon* (*Or-IIIa*)]. The sequence data of wild rice and weedy rice were from Sun et al. (2022), and the sequence of the *japonica* population was obtained from the database http://ricevarmap.ncpgr.cn/. The haplotype network was built based on 14 SNP variations of the *Hd1* Coding sequence (CDS) region with *Or-IIIa* as an outgroup by using the “Median Joining Network” approach implemented in Popart software (Version 1.7).

## Results

### High-latitude weedy rice heading earlier than that of cultivated rice and landrace

**Figure 1:**
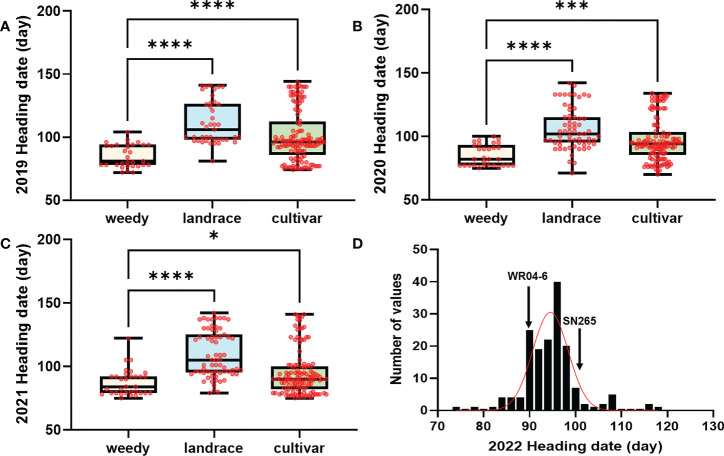
The heading date in the GWAS and RIL population. **(A–C)** The heading date of weedy rice, landrace, and *japonica* cultivar for 2019, 2020, and 2021 in the GWAS population. **(D)** Distribution of the RIL progenies of the weedy rice WR04-6 and *japonica* cultivar SN265. Bars are the maximum and minimum values of samples. Data were analyzed by one-way ANOVA multiple comparisons. ****, ***, and * represent significant differences at P = 0.0001, P = 0.001, and P = 0.05, respectively; red dots indicate samples. The D’Agostino–Pearson test method was used for the normal distribution test. weedy, weedy rice; landrace, japonica landrace; cultivar, modern japonica cultivar.

In high-latitude paddy ecosystems, weedy rice tends to heading earlier than cocultivated rice for rapid completion of reproduction. In the present study, we collected 274 accessions from three *japonica* rice ecotypes as a GWAS panel: the high-latitude modern *japonica* cultivar and its accompanied *japonica* weedy rice and *japonica* landrace. The heading date of this panel was investigated for three consecutive years (2019, 2020, and 2021) as the phenotype for GWAS. In three ecotypes, weedy rice (average 85 days) exhibited significantly earlier heading than cultivated rice (average 96 days) and landrace (average 106 days) (**Figures 1A–C**).

### Dissecting the genetic basis of the weedy rice early heading by genome-wide association study

**Figure 2:**
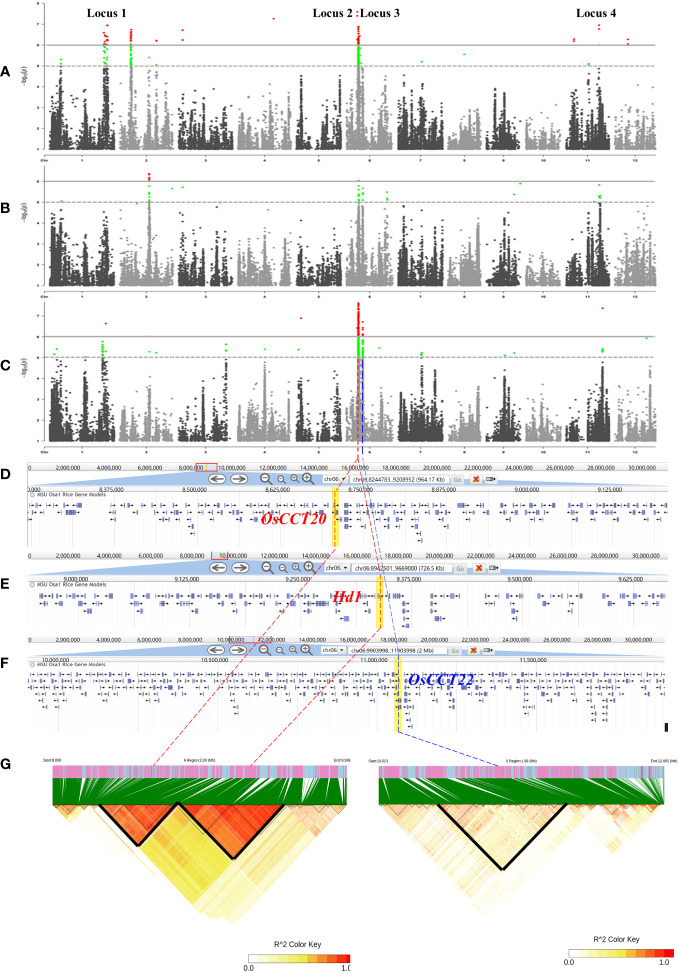
Results of the genome-wide association study. **(A–C)** Manhattan plot of 274 accessions in 2019, 2020, and 2021. Gray lines indicate threshold lines by 10^-5^ and 10^-6^; red and green dots mean SNPs above the threshold line. **(D–F)** Annotation of genes that are in highly linkage disequilibrium block **(G)** linkage disequilibrium block analysis of 2MB region surrounding the lead SNPs, highly linkage disequilibrium blocks are displayed in bold lines.

In the present *japonica* rice panel, the heading date exhibits significant variations. To find quantitative trait nucleotides (QTNs) that are significantly associated with this phenotypic variation, the heading date was investigated for three consecutive years (2019, 2020, and 2021). The Manhattan plots of the GWAS (using a linear mixed model and the first two principal components of PCA as covariates) showed that the four strongest associated signals (Locus 1, Locus 2, Locus 3, and Locus 4) were stable and detected for at least 2 years (**Figures 2A–C**). The specific GWAS results and the locus distribution of ecotype information are displayed in **Table 1**.

**Table 1:** Results of the genome-wide association analysis.

Locus	Chr	Lead SNP (bp)	-log_10_(*P*)	Year	Variation	Weedy	Landrace	Cultivar
**Locus 1**	1	35,416,622	5.77	2021	T/C	0.455	0.689	0.903
	1	36,347,597	6.59	2019	G/GA	1.000	0.770	0.903
	2	7,738,314	6.73	2019	T/A	0.969	0.851	0.968
	2	19,840,643	6.32	2020	G/A	0.000	0.172	0.084
**Locus 2**	6	8,621,625	7.4	2019	A/G	0.879	0.598	0.617
	6	8,718,748	8.1	2021	A/G	0.879	0.621	0.643
	6	8,721,885	6.02	2020	A/C	0.121	0.299	0.425
**Locus 3**	6	10,973,241	4.87	2019	C/T	0.849	0.736	0.636
	6	10,962,053	5.66	2020	T/A	0.819	0.759	0.630
	6	10,958,625	6.71	2021	A/G	0.030	0.770	0.266
**Locus 4**	11	22,255,595	6.95	2019	G/A	0.969	0.805	0.864
	11	22,305,044	5.83	2020	A/G	1.000	0.851	0.877
	11	24,600,828	7.40	2021	ATG/A	0.848	0.471	0.623

The right three columns represent the distribution of the lead SNP variant in the weedy, landrace, and modern cultivar. Chr, Chromosome; weedy, weedy rice; landrace, *japonica* landrace; cultivar, modern *japonica* cultivar.

**Figure 3:**
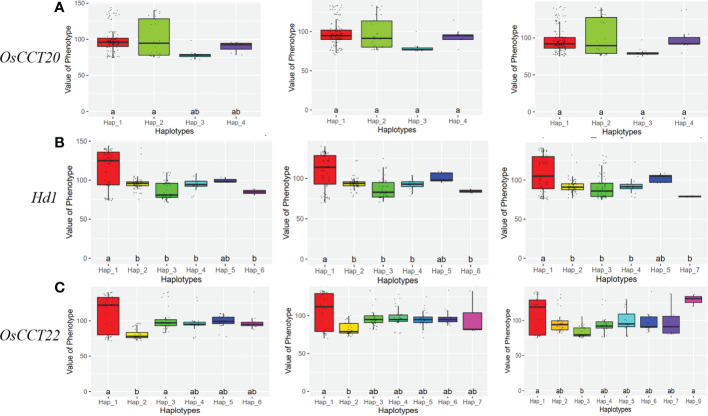
Boxplots of the heading date phenotype for different haplotypes of candidate genes. **(A)** A 3-year heading date box for the different haplotypes of *OsCCT20*. **(B)** A 3-year heading date box for the different haplotypes of *Hd1*. **(C)** A 3-year heading date box for the different haplotypes of *OsCCT22.* Duncan’s test at p ranks the phenotypic differences. The letter a and b are ranked by Duncan’s test at p<0.05.

Considering the following QTL analysis in this study and the background knowledge on the known heading date genes in rice, Locus 2 and Locus 3 on chromosome 6 were the main objects of interest for further analysis. We expanded the 2MB physical distance surrounding the lead SNP of Locus 2 and Locus 3 to find highly LD regions. We finally detected two LD blocks in Locus 2 and one LD block in Locus 3 that involved 361 and 85 genes, respectively (**Figure 2G**). Based on the candidate gene association analysis, we further narrowed down the candidate genes to 24 in Locus 2 and 38 in Locus 3 (**Supplementary Table S4**). On the other hand, three genes according to the annotation (http://rice.uga.edu/), *OsCCT20*, *OsCCT21* (*Hd1*), and *OsCCT22*, within the three LD blocks were considered as candidate causal genes due to all of them being CCT family members that were reported to have important roles in flowering regulation (Zhang et al., 2021) (**Figures 2D–F**). However, considering the candidate gene association analysis, different haplotypes of OsCCT20 did not show significant differences in the heading date. Finally, *OsCCT21* (*Hd1*) and *OsCCT22* were speculated to be the causal genes for responding to the natural variation of the heading date in Locus 2 and Locus 3 (**Figures 3A–C**).

### QTL mapping for the heading date differences between WR04-6 and SN265

**Figure 4:**
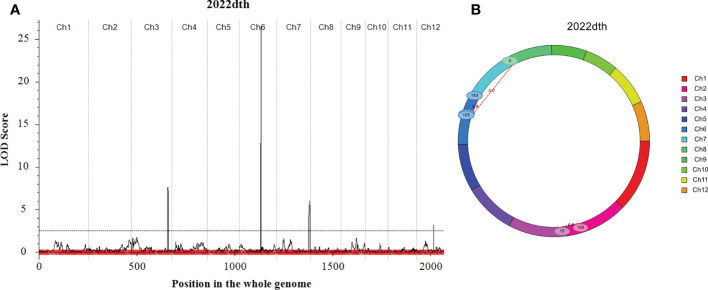
QTL mapping results of the heading date. **(A)** Additive effect for the heading date calculated by the ICIM-ADD model. **(B)** The epistatic effect for the heading date calculated by the ICIM-EPI model. The threshold line was set with an LOD score ≥ 2.5.

**Figure 5:**
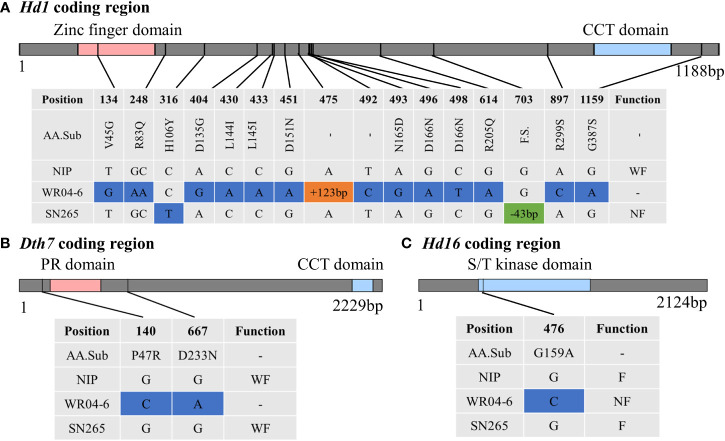
Genotype analysis between the weedy rice WR04-6 and *japonica* cultivar SN265. **(A)** The variation of *Hd1* between WR04-6 and SN265 with Nipponbare genotype as a reference in the coding region. **(B)** The variation of *Dth7* between WR04-6 and SN265 with Nipponbare genotype as a reference in the coding region. **(C)** The variation of *Hd16* between WR04-6 and SN265 with Nipponbare genotype as a reference in the coding region. The blue box indicates a single base variation; the orange and green boxes represent insertion and deletion, respectively. F, Functional; WF, Weak functional; NF, Non-functional. The gray top bar represents the coding region; the colorful region means the domain of the gene.

*Japonica* rice SN265 with a high yield advantage was recognized as a “super rice” by the Chinese Ministry of Agriculture, and its accompanied weedy rice (named WR04-6) showed 10–15 days earlier heading than SN265. The heading date showed continuous variation in the RIL population from 74 days to 123 days, but not following a normal distribution (k^2^ = 30.19) (**Figure 1D**). We crossed SN265 with WR04-6 to develop an RIL population for detecting the genetic basis of these differences in the heading date by QTL mapping. We applied the ICIM-ADD model for additive-effects QTL mapping and found four QTLs above the threshold, as shown in **Figure 4A**; they were distributed on chromosomes 3, 6, 7, and 12 (**Table 2**). Among them, we noticed that three genes within the three QTL genomic regions were reported before: *Hd1/OsCCT21* (Chr6), *Dth7* (Chr7), and *Hd16* (Chr3). Among them, *Hd1* can explain the largest phenotypic variation (PVE = 35.9%); the phenotypic variance explained (PVE) of *Hd16* is 8.3% and that of *Dth7* is 6.1%. Moreover, the additive effect of *Hd1* is 6.3111, while the additive effect of *Hd16* and *Dth7* is -2.1566 and -1.83, respectively. We further analyzed the genotype of the three genes between the two parents, weedy rice WR04-6 and *japonica* cultivar SN265, to confirm the specific variants (**Figures 5A–C**). We found that the genotype of *Hd1* in WR04-6 in the coding region is different from that of SN265 and Nipponbare (reference genome). The changed base located in 134 coding regions in the zinc finger domain of *Hd1* caused a non-synonymous substitution from valine to glycine, which may cause the functional variation of *Hd1* between SN265 and WR04-6 (**Figure 5A**). Considering the GWAS results of the heading date in the *japonica* panel of the present study, we have reason to believe that *OsCCT21 (Hd1*) is the common cause of the early-heading feature in weedy rice.

**Table 2:** RIL QTL mapping result by ICIM-ADD.

Trait Name	Chr	Position(cM)	L-Position(bp)	R-Position(bp)	LOD	PVE (%)	Add	Known Gene	Reference
2022dth	3	184.7	32,196,269	33,001,571	7.6207	8.316	-2.1566	*Hd16*	Hori et al., 2013
2022dth	6	110.5	9,676,469	9,035,697	26.4224	35.9071	6.3111	*Hd1*	Yano et al., 2000
2022dth	7	169	28,914,563	29,617,569	5.9928	6.1069	-1.8338	*Dth7*	Yan et al., 2013
2022dth	12	85.6	21,255,481	21,603,023	3.1934	3.1561	-1.3223	–	–

How *Hd1* of weedy rice evolves is another important scientific issue. Therefore, we established the genetic relationship between weedy rice and other ecotypes of *japonica* through the haplotype network. As shown in **Supplementary Figure S4**, weedy rice has four haplotypes, *Hd1*:Hap2, Hap3, Hap6, and Hap7. Most of the weedy rice (76.9%) is Hap7, which is exclusive to weedy rice and derived from Hap4, the major haplotype shared by temperate *japonica*, tropical *japonica*, and *japonica* intermediate. This result implies that selection pressure on weedy rice resulted in its *Hd1* diverging from cultivated rice under long-term natural selection, which is further evidence of the genetic contribution of *Hd1* in the early heading of weedy rice.

The genotype of *Dth7* and *Hd16* in minor effect QTL genomic regions also showed functional variations between WR04-6 and SN265. *Dth7* has two non-synonymous substitutions in the WR04-6 coding region nearby the PR domain; although this haplotype of *Dth7* has been reported before, its function is still unknown (Gao et al., 2014; Li et al., 2015; Li et al., 2018). A non-synonymous substitution in the WR04-6 S/K kinase domain caused the loss of function of *Hd16* (Kwon et al., 2014) (**Figures 5B, C**). The other QTLs located on chromosome 12 were new unknown QTLs for the rice heading date reported by this study. In addition, we found three gene pairs that may regulate the heading date by epistasis effect interaction based on the ICIM-EPI (**Figure 4B**). Interestingly, one epistasis gene pair occurred between *Hd1* and another locus; the specific details are displayed in **Supplementary Table S5**.

### Breeding early-heading high-yield *Japonica* rice by applying early-heading genes from weedy rice

In developing the RIL population using the weedy rice WR04-6 and the super-high-yielding *japonica* rice SN265, we also performed genetic improvement practices of early-heading lines. From the F_3_ to the F_8_ generation, individuals with early heading and good comprehensive traits were selected as the selection targets in each generation. Finally, six genetically stable lines with different genetic backgrounds were successfully bred, and their heading dates ranged from 81 to 92 days, at least 10 days earlier than that of the *japonica* parent SN265. After detecting the genotype, we found that all of the fragments of Locus 2 and Locus 3 on chromosome 6 of these six lines were derived from the weedy rice parent WR04-6 (**Supplementary Figure S3**). We then cultivated the six lines in higher latitudes, Wuchang, Heilongjiang province (127°16′E, 44°93′N), and confirmed that they could safely head. Thus, our practice of this genetic improvement using weedy rice as a genetic resource has successfully expanded the planting range of super *japonica* rice northward by about 400 km. We also developed molecular markers for this early-heading interval that combined with the early-heading genetic resources of weedy rice will greatly contribute to breeding the early-heading *japonica* rice.

## Discussion

### Molecular genetic basis of early-heading characteristics in weedy rice

In high-latitude paddy ecosystems, weedy rice tends to head early than cocultivated rice for rapid completion of reproduction. In this study, we performed a GWAS and QTL mapping to reveal the genetic basis of the early-heading feature of weedy rice. Our research has found that *Hd1* and *OsCCT22* may be responsible for the heading date variations in the natural population by GWAS (Takahashi et al., 2009; Zhang et al., 2021). Furthermore, *Hd1*, *Dth7*, and *Hd16* were colocated using the ICIM-ADD model in the RIL population, and the three genes do have variations between parents. We also found the epistatic effect of *Hd1* for another QTL by the ICIM-EPI model. Considering the GWAS results with QTL mapping, we believe that *Hd1* is the common cause of the early-heading feature in weedy rice. Our findings provide a new perspective for studying the molecular genetic mechanisms and regulatory networks governing the heading date in rice.

*Hd1* contains the B-box zinc finger domain and CCT domain, which is the first cloned gene that can regulate the heading in rice from Nipponbare (Yano et al., 1997); it may be a selection target in the process of domestication of flowering diversity of cultivated rice (Takahashi and Shimamoto, 2011). In our study, we found a new haplotype of *Hd1* in WR04-6, which contains a new non-synonymous substitution in the B-box binding domain. Based on available data, we speculate that this new haplotype is functional because the conserved CCT domain has no variation in WR04-6, and the additive effect of *Hd1* is 6.311 in the RIL population, which means that the early-heading characteristic comes from the weedy rice WR04-6 (Takahashi et al., 2009). In addition, this substitution in the B-box binding domain may change the protein interaction and affect the other aspect of rice growth. More importantly, this single base substitution variation only exists in weedy rice and is not detected in other accessions, but we did not validate the activity of the *Hd1* protein of weedy rice.

### Variant genes in weedy rice change the complex regulatory network of the heading date

The flowering time of rice is regulated by plenty of genes; different combinations of these allelic genes determine the rice adaptability and regional distribution (Ebana et al., 2011; Guo et al., 2013; Lee and An, 2015; Brambilla et al., 2017). *Hd1* has a basic function of promoting the expression of florigen genes *Hd3a*/*RFT1* and heading regardless of the day length (Zong et al., 2021). *Hd1* interacts with the *Ghd7* (*OsCCT26*) CCT domain to form a complex that represses the rice heading. The *Dth8/Hd1* complex binds to the promoter of *Ghd7* (*OsCCT26*) to form a ternary complex (Lin et al., 2003; Nemoto et al., 2016; Du et al., 2017; Wang et al., 2019; Zhang B. et al., 2019; Zhang Z. et al., 2019; Zong et al., 2021). *Dth7* (*OsCCT28*) is a major gene regulating the flowering time in the northeast of China, which also regulates the plant height and grains per panicle. *Dth7* acts downstream of phyB, inhibiting the expression of *Ehd1* (upstream regulator of rice florigen genes *Hd3a* and *RFT1*), thereby delaying flowering under day length (Liu et al., 2013; Gao et al., 2014; Liu et al., 2021). We speculate that the *Dth7* haplotype found in WR04-6 is non-functional because the additive effect of *Dth7* is -1.8338 in the RIL population (**Table 2**); the non-functional *Dth7* leads to the early heading of weedy rice by reduced suppression of *Ehd1* and promotes the expression of florigen genes *Hd3a*/*RFT1* in the shoot apical meristem. The non-functional *Dth7* reduced the competition for *Hd1*, which means that *Hd1* will play a greater role in promoting the heading under long day-length conditions. *Hd16* can phosphorylate *Ghd7* (*OsCCT26*), which contributes to the later heading in *japonica* rice under LD conditions. *Hd16* can also interact with and phosphorylate *Dth7*, although the underlying mechanism is still unknown (Kwon et al., 2015). The genetic interactions between *Ghd7* and *Dth7* and between *Dth7* and *Hd16* are also involved in the heading under LD conditions (Shibaya et al., 2011). The non-functional *Hd16* in weedy rice will reduce the expression of *Dth7* and *Ghd7*, thereby enhancing the expression of *Ehd1* and promoting the heading. Variations found in weedy rice change the complex regulatory network of the heading date and other aspects of rice growth. Based on the above research, we can conclude that the early heading of weedy rice results from natural selection and competition with cultivated rice; however, how selections shape the early-heading loci in the weedy rice genome is still unknown. We uncovered this scientific question through the GWAS and QTL mapping in this study.

### Weedy rice as a gene resource for improving cultivated rice

As an important ecological characteristic, the heading date plays a decisive role in the rice breeding practice. It affects cultivation environments, yield, rice quality, nutritional value, and other traits; suitable heading dates will maximize the use of light, heat, and other ecological resources in the local ecological environment (Brambilla et al., 2017). The early heading is a necessary feature for weedy rice to survive. Under long-term natural selection, the elite dominant allele of the early heading in weedy rice has been fixed. It is easy to reproduce naturally in the paddy field and breed offspring for weedy rice (Xia et al., 2011; Sun et al., 2013). Therefore, exploring early-heading gene resources of weedy rice is of great value for cultivating the early-heading cultivated rice.

Weedy rice can be an excellent genetic resource for its large number of elite characteristics, the most important is that weedy rice has no reproductive isolation with cultivars. Thus, these elite genes can be applied in the breeding practice and improvement programs for cultivars by Molecular marker-assisted selection (MAS). The genetic diversity of cultivated rice has decreased yearly due to the single breeding objective, so the genetic resource from weedy rice is very precious (Ebana et al., 2011). Applying the early-heading genes from weedy rice makes more elite cultivated varieties that can be planted in more expansive areas (Imaizumi et al., 2021). In the present study, we also developed several molecular markers to assist the selection of the early-heading varieties and expand the rice-suitable cultivation area northward.

Besides the early-heading characteristics, weedy rice still has many other fine biological characteristics that can be used in breeding. All these make weedy rice more competitive than the cultivated rice. Weedy rice is a precious genetic material for cultivated rice that is reported to be a hidden gold mine in the paddy field (Tang et al., 2011; Qiu et al., 2017; Sun et al., 2019; Wu et al., 2022). Especially in high latitudes where wild rice is difficult to apply, weedy rice might be the best choice to expand the genetic diversity of *japonica* rice.

## Data availability statement

The original contributions presented in the study are included in the article/**Supplementary Material**. Further inquiries can be directed to the corresponding authors.

## Author contributions

JS and WC designed the study. ZL, RG, JC, and XZ investigated the heading date trait. ZL and RG performed data statistical analysis. XY and JS performed GWAS analyses. ZL, XY, and CL carried out candidate gene association analysis. XmZ and PY performed searching candidate genes/QTLs. ZL wrote the paper. JS revised the manuscript. All authors read and approved the final manuscript.

## Funding

This work was supported by the Liaoning Applied Basic Research Program (2022JH2/101300172), and the Support Program for Young Scientific and Technological Innovation Talents of Shenyang, China (No. RC210408).

## Conflict of interest

The authors declare that the research was conducted in the absence of any commercial or financial relationships that could be construed as a potential conflict of interest.

## Publisher’s note

All claims expressed in this article are solely those of the authors and do not necessarily represent those of their affiliated organizations, or those of the publisher, the editors and the reviewers. Any product that may be evaluated in this article, or claim that may be made by its manufacturer, is not guaranteed or endorsed by the publisher.
