# Supplementary material for: Genetic basis of the early heading of high-latitude weedy rice
Source: Front Plant Sci. 2022 Dec 5;13:1059197. doi: 10.3389/fpls.2022.1059197 (PMC9760980; doi:10.3389/fpls.2022.1059197)
Supplement: Supplementary file 1 [file DataSheet_1.docx]

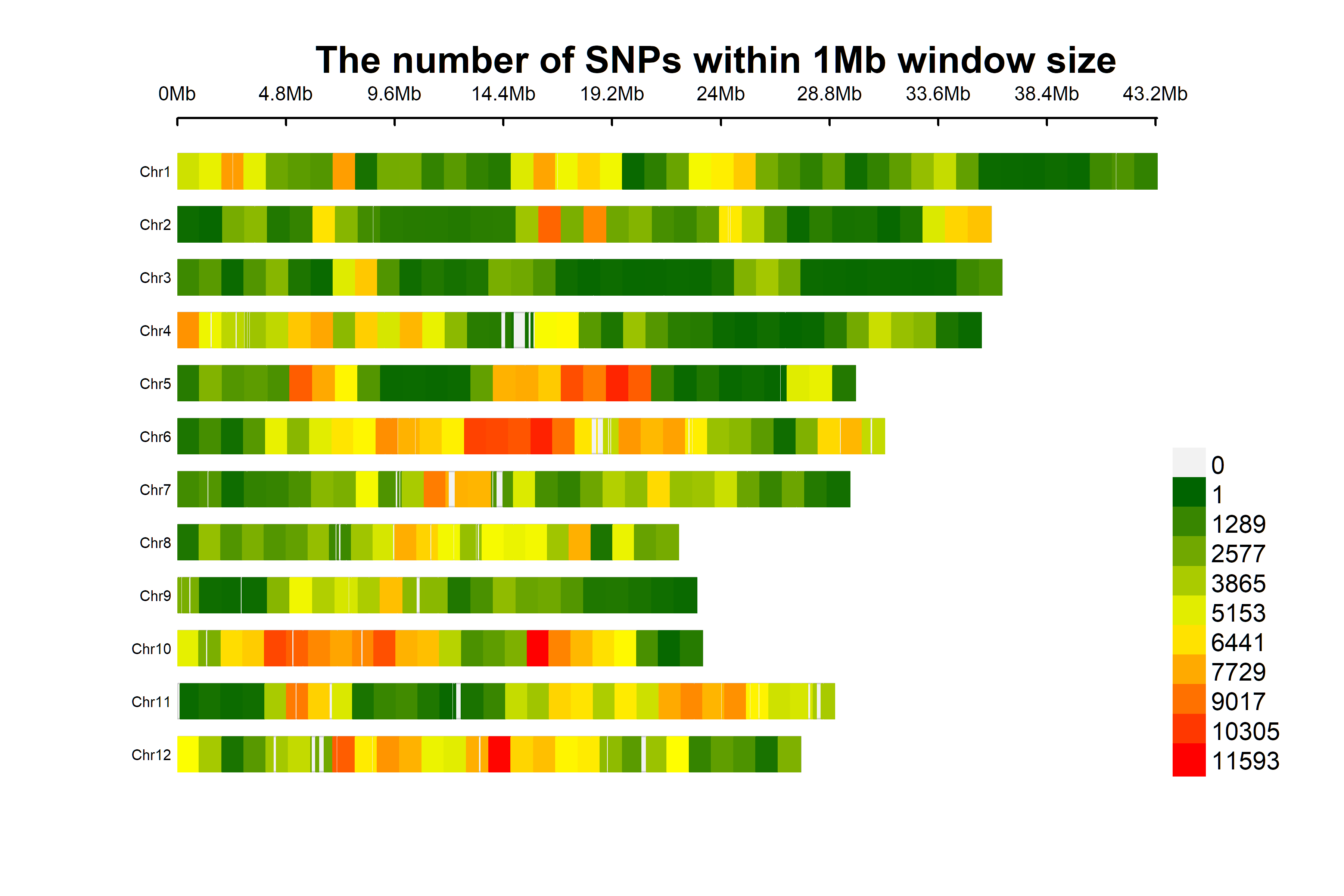


**Supplementary Figure S1** | Graph of SNPs distribution on 12 chromosomes of GWAS population.

F, Functional; WF, Weak Functional; NF, Nonfunctional, UK, Unknown

LN-C, Liaoning cultivar; LN-W, Liaoning weedy;

JL-C, Jilin cultivar; JL-W, Jilin weedy;

HLJ-C, Heilongjiang cultivar; HLJ-W, Heilongjiang weedy


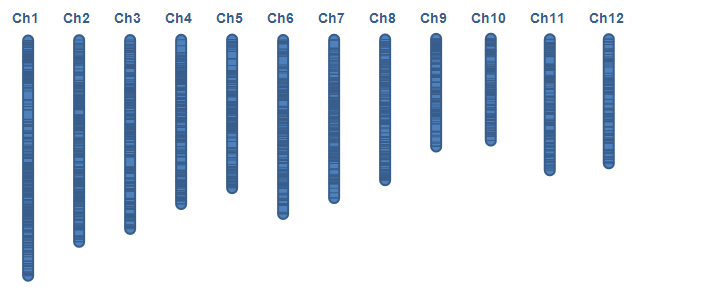


**Supplementary Figure S2**| Graph of SNPs distribution on 12 chromosomes of RILs derived from weedy rice WR04-6 and *japonica* cultivar SN265

**Supplementary Figure S3**| Graph of MAS breeding program. The black box represents the genotype of weedy rice WR04-6, and the white box indicates the genotype of super-high yielding japonica cultivar SN265; Locus 2 and Locus 3 are consistent with GWAS results.


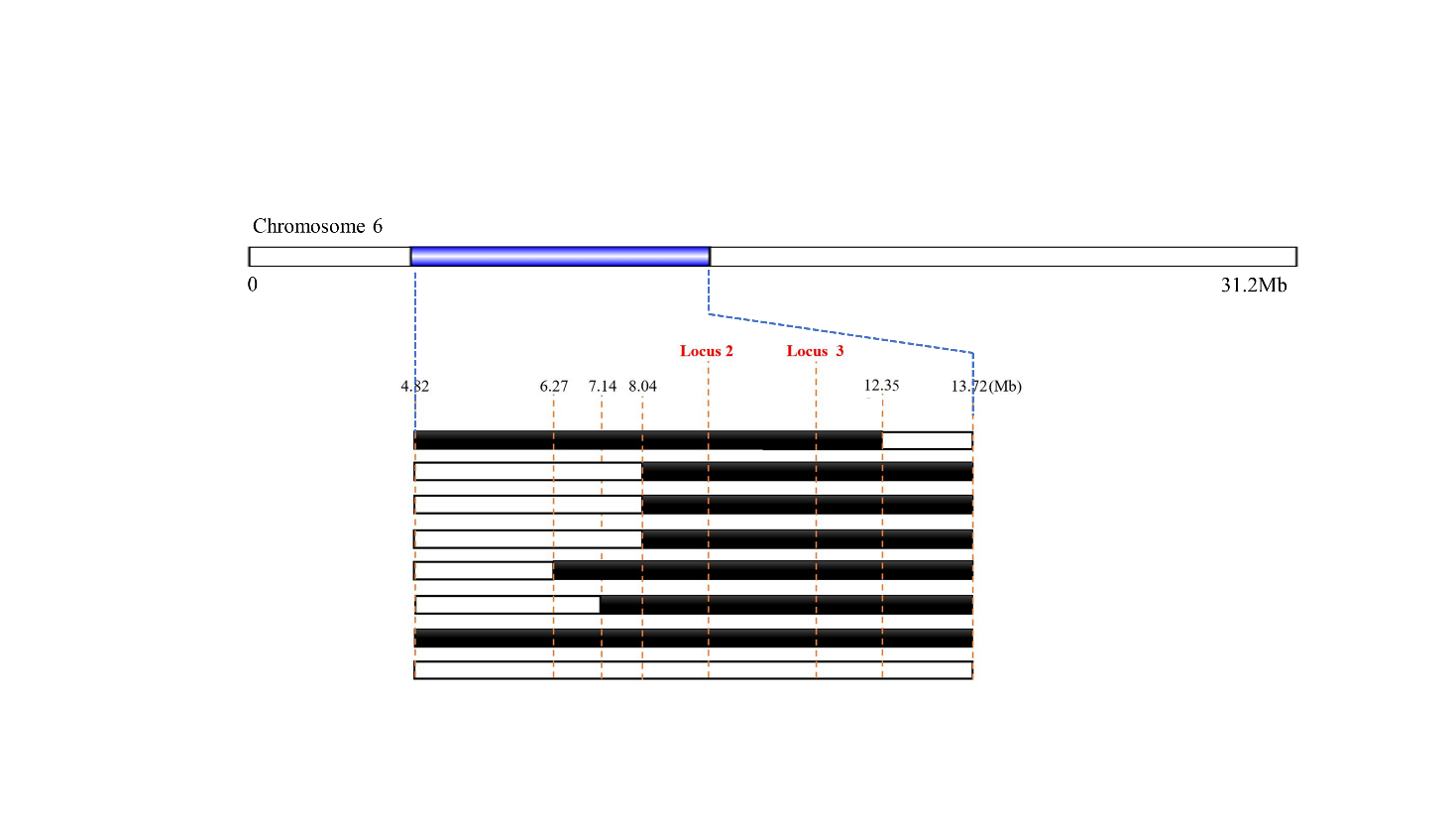


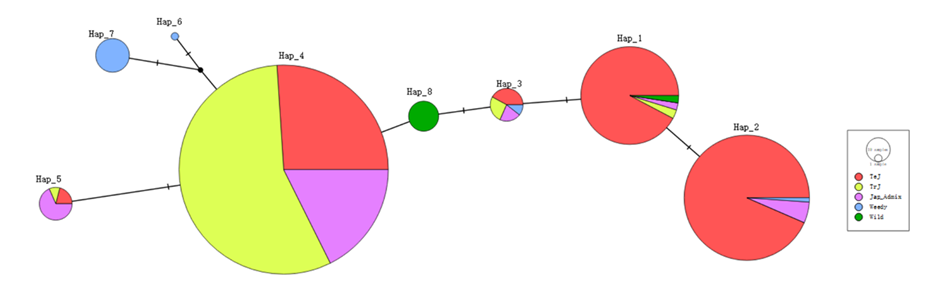


**Supplementary Figure S4**| Haplotype network of *Hd1* for five ecotypes, wild rice was used as an outgroup. circle size represents the sample size for a given haplotype. Black bars on the lines indicate mutational steps between haplotypes. Different colors represent different ecotypes. TeJ, Temperate *Japonica*; TrJ, Tropical *Japonica*; Jap_Admix, ***Japonica* Intermediate;** Weedy, weedy rice; Wild, wild rice
